# Effect of Unna's boot on venous ulcer healing: a systematic review and meta-analysis

**DOI:** 10.1590/1980-220X-REEUSP-2023-0397en

**Published:** 2024-09-02

**Authors:** Fernanda Peixoto Cordova, Ana Claudia Furhmann, Andreia Cristina Feitosa do Carmo, Eduardo Nunes Vales, Diego Henrique Terra, Bárbara Uuritz da Silva, Diani de Oliveira Machado, Amália de Fátima Lucena, Lisiane Manganelli Girardi Paskulin

**Affiliations:** 1 Universidade Federal do Rio Grande do Sul, Escola de Enfermagem, Porto Alegre, RS, Brazil.; 2 Universidade Federal de São Paulo, Biblioteca, São Paulo, SP, Brazil.; 3 Universidade Federal de Ciências da Saúde, Faculdade de Medicina, Porto Alegre, RS, Brazil; 4 Centro Universitário Ritter dos Reis, Escola de Enfermagem, Porto Alegre, RS, Brazil.; 5 Hospital Nossa Senhora da Conceição, Porto Alegre, RS, Brazil.

**Keywords:** Varicose Ulcer, Compression Bandages, Stockings, Compression, Systematic Review, Meta-Analysis, Úlcera Varicosa, Vendajes de Compresión, Medias de Compresión, Revisión Sistemática, Metaanálisis

## Abstract

**Objective:**

To analyze the effect of Unna’s Boot on the healing of venous ulcers compared to other therapies.

**Methods:**

Systematic Review carried out in the databases *Scopus, Embase, Cochrane Library, Web of Science, PubMed, Cumulative Index of Nursing and Allied Health Literature, Latin American and Caribbean Literature in Health Sciences, and grey literature*. Population – adult patients with venous ulcers; Intervention- Unna’s Boot (UB); Control – other compression therapies (CT); Outcome- healing; Designs- randomized clinical trial, cohort study, and case control, published from 2001 to 2024. The effect of the intervention, risk of bias, and quality of evidence were evaluated. Registered with PROSPERO (CRD42021290077).

**Results:**

A total of 39 studies were included, with 5.151 patients. The majority (71.8%) were randomized controlled trials (RCT). UB was used as intervention/control in eight studies. When comparing CTs, only 1 study with UB showed a superior effect (p < .001) in healing, compared with high compression elastic bandage. In the quality of evidence analysis, 27 studies were assessed as having a high risk of bias.

**Conclusion:**

No superiority of UB was found in the healing of venous ulcers when compared to other CTs.

## RESUMEN

**Objetivo:**

Analizar el efecto de la Bota de Unna en la cicatrización de úlceras venosas en comparación con otras terapias.

**Métodos:**

Revisión sistemática realizada en las bases de datos *Scopus, Embase, Biblioteca Cochrane, Web de la Ciencia, PubMed, Índice acumulativo de literatura de enfermería y salud afines*, Literatura Latinoamericana y del Caribe en Ciencias de la Salud, y literatura gris. Población – pacientes adultos con úlceras venosas; Intervención- Bota de Unna (BU); Control: otras terapias de compresión (TC); Resultado- curación; Diseños: ensayo clínico aleatorizado, estudio de cohorte y casos y controles, publicado del 2002 al 2023. Se evaluaron el efecto de la intervención, el riesgo de sesgo y la calidad de la evidencia. Registrada en PRÓSPERO (CRD42021290077).

**Resultados:**

Se incluyeron 39 estudios, con 5.151 pacientes. La mayoría (71,8%) fueron ensayos controlados aleatorios (ECA). La BU se utilizó como intervención/control en ocho estudios. Al comparar TC, sólo 1 estudio con BU mostró un efecto superior (p < .001) en la curación, en comparación con el vendaje elástico de alta compresión. En el análisis de la calidad de la evidencia, se evaluó que 27 estudios tenían un alto riesgo de sesgo.

**Conclusión:**

No se encontró superioridad de la BU en la curación de úlceras venosas en comparación con otras TC.

## INTRODUCTION

Venous ulcer (VU) is a serious outcome of Chronic Venous Insufficiency (CVI)^(1)^, accounting for 85% of cases of chronic leg ulcers with a prevalence between 1.5 and 3% in the population^(2,3)^. The gold standard in VU treatment is the application of compression therapy (CT)^(1,3)^, which consists of applying external compression to the leg to promote venous return and reabsorption of edema, reduce hypertension and venous stasis, contributing to healing and reducing VU recurrence^(1,4)^.

CT can be static or dynamic. Static therapy is produced by elastic or inelastic action of bandages and compression stockings. Dynamic therapy is performed by intermittent pneumatic compression^(1,2)^. Among static CTs, there are several types, classified according to the type and number of components and/or layers used, effect, and levels of compression applied^(1,2)^.

In this context, Systematic Reviews (SR) were conducted addressing different CTs. In a 2012 study of 59 randomized controlled trials (RCTs) (n = 4,321), it was concluded that CT increases VU healing rates compared to the use of non-graded compression bandages, i.e. those made of crepe, Rayon^®^, or mixed synthetic fabric. Furthermore, it was observed that multicomponent and multilayer compression systems, especially those with elastic bandages, were more effective than systems with a single component or consisting mainly of inelastic bandages. No significant evidence was found for healing among other therapies, such as the Unna Boot (UB), compared with four-layer bandages, elastic stockings, and adjustable inelastic stockings^(5)^.

Another SR involving 14 RCTs (n = 1,391 patients) evaluated the effect of short-stretch bandages, four-layer bandages, and UB compared to the absence of compression. The results demonstrated faster healing when using any bandage compared to the absence of compression and indicated a possible improvement in some aspects of quality of life and pain^(6)^.

The results of the aforementioned SR indicate that the use of CT is more effective for healing than not using it. However, due to the existence of different types of CT, there is no global consensus regarding the use of a specific type^(1,2,5,7)^. In the United Kingdom, four-layer bandaging is widely applied, whilst in continental Europe and Australia short-stretch bandaging is a more frequent practice. In Brazil and the United States, UB is the most common^(5,8)^. Nevertheless, in Brazil, for the treatment of people with VU there is still a wide use of bandages with no compression classification^(9)^, that is, bandages made of ordinary fabrics.

The UB, invented in 1885, consists of an inelastic compression bandage that acts by increasing venous pressure during muscle contraction, especially during ambulation^(10,11)^. An SR carried out with 08 studies (n = 643 patients) aimed to determine the effectiveness of UB in the treatment of VU, comparing UB to other types of CT, associated or not with primary coverage. In the meta-analysis, regarding the healing rate, no difference was found between the therapies (OR 0.45), and regarding the healing time, UB had slower healing^(12)^.

As already mentioned, although UB was invented a long time ago and despite different types of compression therapies, it is still widely used in the treatment of VU in the global context, mainly in Brazil, in Primary Health Care services. In this context, despite empirical clinical observation demonstrates apparent satisfactory effects on healing, much of the scientific evidence that compared UB with other therapies was not robust enough to identify and justify its wide use and measure whether the effect perceived in clinical practice is statistically significant. Furthermore, these studies are not recent. In view of this, it is believed that a new evaluation of studies regarding the use of UB can contribute to the improvement of clinical management and assist in the formulation of more assertive guidelines and care strategies, as well as the implementation of effective inputs for patient care, mainly in Brazil, in Primary Health Care services, the gateway and main place of care for users with VU. Therefore, this study had the following research question: What is the effect of Unna’s Boot on the healing of venous ulcers compared to other compression therapies?

## METHODS

### Protocol and Registration

This is a Systematic Review with Meta-Analysis prepared according to the Cochrane^(13)^ recommendations, presented in accordance with the Preferred Reporting Items for Systematic Reviews and Meta-Analyses (PRISMA)^(14,15)^. The study protocol was registered in PROSPERO (CRD42021290077), with the title: *Effect of Unna Boot on healing, pain, edema and quality of life in patients with venous ulcers: a Systematic Review* ^(16)^.

### Eligibility Criteria

The PICOS mnemonic was: Population – adult patients with venous ulcers; Intervention- Unna Boot (UB); Control – other compression therapies (CT); Outcome- healing; Designs- randomized clinical trial, cohort study, and case control, published from 2002 to 2023. The intervention of interest was CT UB. The comparator was the other CTs^(1,2)^. Studies carried out with adults with VUs undergoing CT treatment were included, with comparisons between different therapies and CT with bandages without compression classification, referred to as usual care. Studies published between November 2001 and January 2024, available in full, in Portuguese, English and Spanish, were included. Regarding the design, RCTs were inserted to evaluate the beneficial effects of the treatment, complemented by results from observational studies (cohort and case-control studies). The studies included presented healing as both a primary and secondary outcome. Observational studies presenting only one intervention group were included, as long as that intervention was UB.

Studies that compared the effect of pneumatic CT with other therapies were excluded, with the exception of one that included UB as one of the intervention CTs^(17)^. Studies with co-interventions associated with CT, such as surgical and invasive procedures, pharmacological treatments and dressings, were not included, to reduce interference with the effect of the analyzed outcomes.

### Information Sources

The searches were carried out in January 2024, in the databases: Scopus, Embase, Cochrane Library, Web of Science, PubMed, Cumulative Index of Nursing and Allied Health Literature (CINAHL Complete*)*, Latin American and Caribbean Literature in Health Sciences (LILACS), as well as in grey literature databases – grey literature (opengrey.org) and in the Bank of Theses and Dissertations of the Coordination for the Improvement of Higher Education Personnel (CAPES).

### Data Extraction

The development of database search strategies, database searches and recording of findings in the software Rayyan Intelligent Systematic Review^(18)^ were carried out by a librarian experienced in SR. The literature search in the Database of Theses and Dissertations and Open Grey was carried out by two researchers individually, using simple search terms.

The selection of studies was carried out by two researchers independently. Rayyan software was used to identify possible duplications. Afterwards, the titles and abstracts were read, the researchers classified the studies individually, and the tool analyzed the agreement and conflicts among the selections. There was agreement in 32 studies, 17 were conflicting, and five were classified as perhaps. Conflicts were resolved through discussion among the researchers or with the intervention of a third party. After the final selection, the studies were independently evaluated in full.

### Assessment of Study Quality

The Grading of Recommendations Assessment, Development and Evaluation (GRADE) was used to assess the quality of the body of evidence for the outcome under analysis in the studies included. The GRADE System defines the certainty of a body of evidence with respect to the extent to which one can be certain that an estimate of effect or association is close to the actual quantity of specific interest. Assessing the certainty of a body of evidence using GRADE involves consideration of the risk of bias within the study (methodological quality), objectivity of the evidence, heterogeneity, precision of effect estimates, and risk of publication bias^(19)^.

Outcome results from RCTs are initially scored as high quality, while those generated by observational studies start out as low quality. Next, the weighting system is applied to reduce or increase the evidence quality score. Evidence quality is classified into four levels: high, moderate, low, or very low^(13,19)^.

### Statistical Analysis

The information collected from the included studies were: authors, title, year of publication, journal/publication source, country of origin; objectives; design of study; study population; participant inclusion and exclusion criteria; sample; sample description; study location; recruitment; randomization; blinding; intervention; control; follow-up time; outcomes; other reported outcomes; results; limitations; effect measures; interest effect; adverse events; interpretations of results; conclusions. The results were organized in the Excel spreadsheet editor software.

In the analysis of the healing outcome, the results presented regarding the time (weeks) for VU healing (mean and standard deviation); UV healing rate (n and %) during follow-up and area differences (cm^2^) of the initial and final VU (mean and standard deviation) were considered. When evaluating this outcome, it was expected that the VUs would heal in the shortest time possible, that the percentage of VUs healed would be higher or that there would be a significant reduction in the area from the initial to the final VU under the effect of UB, compared to other CTs. Data were organized into subsets: healing rate, time to healing, and differences in the VU area.

### Risk of Bias

In assessing the risk of bias for RCTs, the tool Cochrane Risk of Bias (ROB-2 tool)^(20)^ was used. Cohort and case-control studies were evaluated using the tool Risk Of Bias In Non-randomized Studies – of Interventions (ROBINS-I tool)^(21)^.

Regarding the assessment of publication bias, we did not find a sufficient number of studies to carry out. The Grading of Recommendations Assessment, Development and Evaluation (GRADE) System was used to assess the quality of the body of evidence for outcome^(22)^.

### Meta-Analysis

Pooled intervention effect estimates were carried out using the software RStudio. The meta-analysis of the healing outcome was carried out in subsets, according to the results presented in the studies, described in statistical analysis. For studies presenting measurements as median, interquartile range, minimum and maximum, conversion was carried out to mean and standard deviation, according to the conversion formulas^(23)^.

### Summary of Results

In the descriptive analysis, data from the included studies were displayed in a table, considering the following items: author and year, country and language, journal/source, main objective, study design, total sample. Another table presented intervention, control data, analyzed outcomes, outcome measurement, follow-up time, and main results. As per guidance from check list from PRISMA, the Adverse Events (AE) reported in the included studies were also presented.

For quantitative analysis, studies needed to be homogeneous in terms of population, exposure, comparator, and outcome. In the healing outcome, similar studies were grouped in terms of the existence of a comparison between CT, according to the subsets identified in this outcome. The meta-analysis was carried out with the forest plot in each subset to evaluate the effectiveness of UB intervention on VU healing compared with other compression therapies.

## RESULTS

### Study Selection

**Figure 1:**
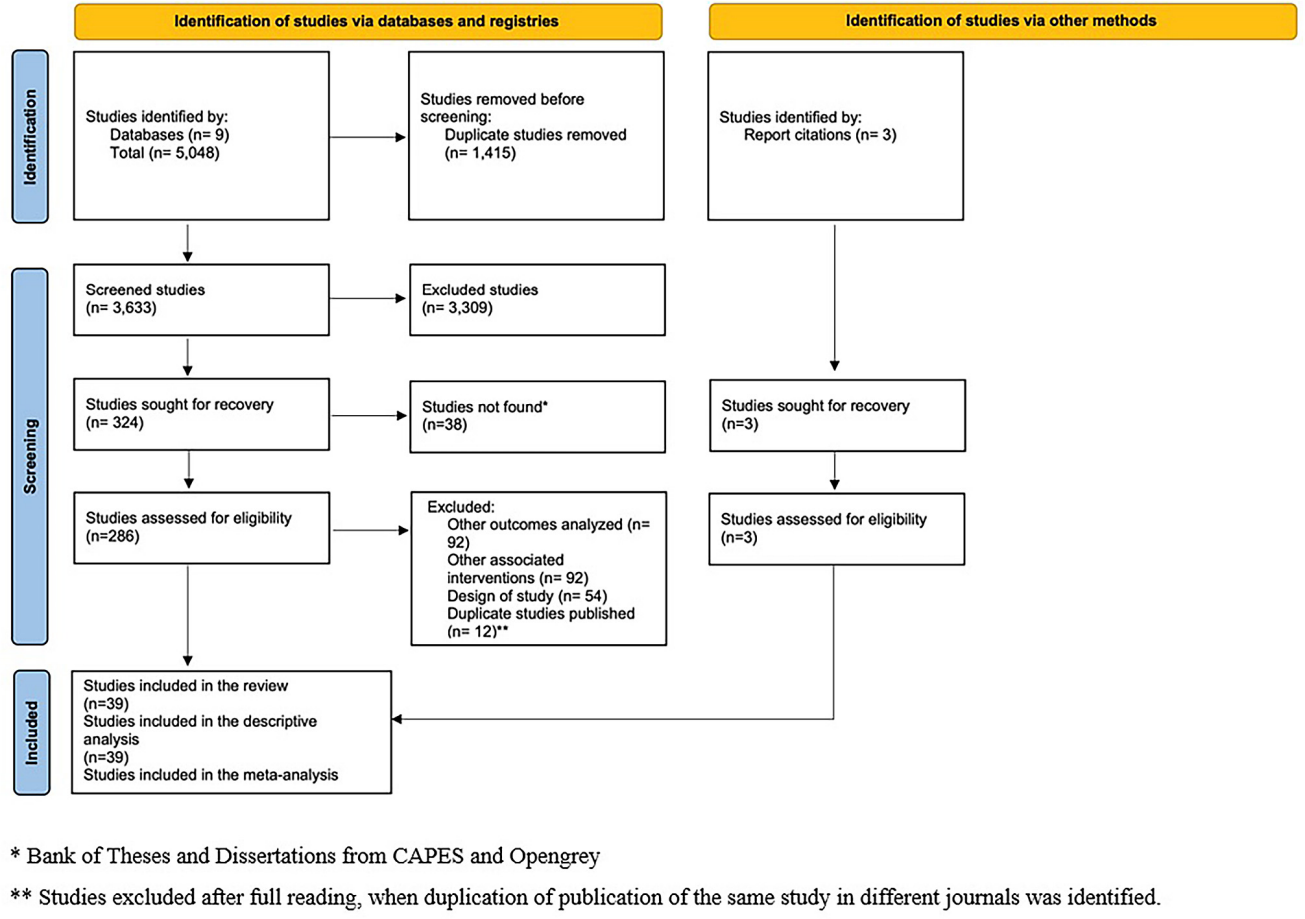
Flowchart of articles selection according to PRISMA 2020.

**Source:** Adapted^(15)^ by authors, 2024.

Database searches resulted in 5,048 studies. Considering the defined criteria, 39 studies were included, as shown in the Flowchart (Figure 1).

Among 39 studies included, the total sample was 5,151 adult patients with VU. Considering the 28 studies that stratified the sample characteristics, the majority were female (54.3%). The studies were published between 2002 and 2023, being more frequent in 2004 and 2014 (n = 5; 12.8% respectively). Within years 2010, 2012 and 2015 there were 3 studies (7.6%) per respective year; in the years 2003, 2008, 2011, 2013, 2019, 2020, 2022 and 2023, 2 studies were published (5.1%) and in the years 2002, 2005, 2007 and 2021, only 1 study (2.5%) was published per year. In the years 2006, 2009, 2016, 2017 and 2018, no studies were published. Regarding the country of origin, the country with the most publications was the United Kingdom (n = 8; 20.5%), followed by Brazil (n = 7; 17.9%), Italy (n = 4; 10,2%); however, adding to the other studies carried out in other countries, it is observed that the majority (n = 25; 64.1%) of the studies were from European countries (Table 1).

**Table 1:** Summary of the characterization of studies included, Porto Alegre (RS), Brazil, 2022.

Variables	N	%
**Year**		
2002	1	2.5
2003	2	5.1
2004	5	12.8
2005	1	2.5
2007	1	2.5
2008	2	5.1
2010	3	7.6
2011	2	5.1
2012	3	7.6
2013	2	5.1
2014	5	12.8
2015	3	7.6
2019	2	5.1
2020	2	5.1
2021	1	2.5
2022	2	5.1
2023	2	5.1
**Country**		
UK	8	20.5
Brazil	7	17.9
Italy	4	10.2
Germany	3	7.6
France	3	7.6
Serbia	3	7.6
Australia	2	5.1
United States of America	2	5.1
Poland	2	5.1
Argentina	1	2.5
Canada	1	2.5
China	1	2.5
Spain	1	2.5
Turkey	1	2.5
**Design of study**		
RCT	14	35.8
Multicenter RCT	12	30.8
Pragmatic multicenter RCT	1	2.6
Cross-over multicenter RCT	1	2.6
Cohort	8	20.5
Retrospective cohort	2	5.1
Case-control	1	2.6
**Sex (28 studies)**	2,247	54.3
Female	1,891	45.7
Male		
**Age (mean/SD) (28 studies)**		
68.6 years (± 6.0)		
**Intervention**		
Unna's Boot	8	20-20.5
4-layers bandage	7	17.9
Elastic stockings	6	15.3
2-layers bandage	5	12.8
High compression bandage	3	7.7
Inelastic stockings	3	7.7
**Control**		
4-layers bandage	9	23.1
Short-stretch bandage	9	23.1
2-layers bandage	8	20.5
Bandage without compression	5	12.8
rating	2	5.1
Unna's Boot		
**Follow-up Time (Median/IQ)**		
Weeks	12	12-24

**Source:** Prepared by the authors, 2024.

Regarding the type of study, 28 (71.8%) were some type of RCT. Regarding the grouping of CTs, UB was used in 20.5% of the investigations as an intervention CT and in 5.1% as a control CT. The median follow-up time in the studies was 12 weeks^(12–24)^ (Table 1).

### Study Results

Chart 1 details the information on the studies included. The way in which the healing outcome was assessed varied greatly between studies and the majority used more than one. The evaluations were: healing rate (n = 30; 76.9%), time to healing (n = 21; 53.8%), and difference in VU area (n = 16; 41.0%) expressed as a percentage of reduction, weekly reduction coefficient and reduction difference between the initial and final VU area.

**Chart 1:** Characteristics of the studies included.

Author year	Study design	Intervention group (IG) (n)	Control group (CG) (n)	Follow-up (weeks)	Summary of main results
Meyer et al., 2002^(24)^	Cohort	High compression elastic bandage (n = 57)	Short-stretch bandage (n = 55)	26	**Healing rate** *p* = .623 IG: 58% (n = 33) CG: 62% (n = 34) Healing time^*^ IG: 10 (95% CI 8–12) CG: 11 (95% CI 9–13)
Meyer et al., 2003^(25)^	Cohort	3-layers (n = 64)	4-layers (n = 69)	56	**Healing rate p = .031** IG: 80% (n = 51) CG: 65% (n = 45) Healing time^*^ *P* = .040 IG: 12 (95% CI 10–15) CG: 16 (95% CI 13–21)
Ukat et al., 2003^(26)^	RCT	4-layers (n = 44)	Short-stretch bandage (n = 45)	12	**Healing rate** IG: 30% (n = 13) CG: 22% (n = 10) Healing time^*^ *p* = .03 OR 2.9 (95% CI 1.1–7.5)
Franks et al., 2004^(27)^	RCT^♦^	4-layers (n = 74)	2 cohesive layers (n = 82)	24	**Healing rate** *p* = .79 IG: 85% (n = 63) CG: 83% (n = 68)
Iglesias et al., 2004^(28)^	RCT^♦^	4-layers (n = 195)	Short-stretch bandage (n = 192)	24	**Healing rate p = .005** IG: 68% (n = 133) CG: 55% (n = 106) Healing time^*^ *p = .12* IG: 92 (95% CI 71–113) CG: 126 (95% CI 95–157)
Junger et al., 2004a^(29)^	RCT^♦^	Elastic stockings (n = 88)	Short-stretch bandage (n = 90)	12	**Healing rate** IG: 58% (n = 51) CG: 57% (n = 51) Healing time^*^ *p = .80* IG:43 (±18.3) CG: 43.6 (±18.3) **UV area difference** ^**#**^ IG: 67.6% CG: 59%
Junger et al., 2004b^(30)^	RCT^♦^	Elastic stockings (n = 61)	2-layers short-stretch bandage (n = 60)	12	**Healing rate p = .0129** IG: 47.5% (n = 29) CG: 31.7% (n = 19) Healing time^**^ *p* = .0297 IG: 61 (±26) CG: 68 (±25)
Polignano et al., 2004^(31)^	RCT^♦^	4-layers (n = 39)	Unna’s Boot (n = 29)	24	**Healing rate** *p* = .42 IG: 74% (n = 29) CG: 66% (n = 19) Healing time^*^ *p = .13* IG: 51 (95% CI 7–175) CG: 49 (95% CI 7–168) **UV area difference** ^#^ *p* = .30 IG: 100% (95% CI –283.3–100) CG: 100% (95% CI –489.3–100)
Blecken et al., 2005^(32)^	RCT	Inelastic stockings (n = 12)	4-layers (n = 12)	12	**Healing rate p = .0173** IG: HR 0.56 CG: HR 1 **VU area difference^**##**^ p = .0369** IG: 2.93 (±0.6) CG: 2.3 (±0.7)
Millic et al., 2007^(33)^	RCT	3-layers with elastic stockings and medium stretch bandage (n = 75)	2-layers medium-stretch bandage (n = 75)	52	Healing time^**^ *p* < .001 IG: 133 (28–464) CG: 211 (61–438)
Mariani et al., 2008^(34)^	RCT^♦^	Ulcer X Kit (n = 26)	Short-stretch bandage (n = 30)	16	**Healing rate p = .011** GI: 96.2% (n = 25) CG: 70% (n = 21)
Moffatt et al., 2008^(35)^	ECR^♦♦^	2-layers (n = 39)	4-layers (n = 42)	08	**Healing rate before** ^♦♦^ *p = .30* IG: 15.3% (n = 6) CG: 7.1% (n = 3) **UV area difference** ^♦♦#^ p = .88 IG: 27.8% CG: 42.2%
Brizzio et al., 2010^(36)^	RCT	Elastic stockings (n = 28)	Usual care (n = 27)	26	**Healing rate** *p* = .210 IG: 50% (n = 14) CG: 67% (n = 18) Healing time^**^ *p = .942* IG: 68 (±40) CG: 69 (±39)
Millic et al., 2010^(37)^	RCT	Class III elastic stockings (n = 42)	2-layers^Δ^ (n = 46) 3-layers^ΔΔ^ (n = 43)	26	**Healing rate** IG: 25% – GI x GC2 ***p* = .000** CG1: 67% CG2: 74% – CG2 x CG1 ***p* = .0238** Healing time^*^ *p > .05* IG: 12 CG1: 11 CG2: 14
Szewczyk et al., 2010^(38)^	RCT	Class II elastic stockings (n = 15)	2-layers^Δ^ (n = 16) 4-layers^ΔΔ^ (n = 15)	12	**Healing rate** *p > .05* IG: 53.3% (n = 8) CG1: 62.5% (n = 10) CG2: 60% (n = 9) **VU area difference** ^**##**^ **p < .001** IG: .44 CG1: .55 CG2: .63
Harisson et al., 2011^(39)^	RCT^♦^	4-layers (n = 215)	Short-stretch bandage (n = 209)	12	Healing time^**^ *p = .98* IG: 62 (95% CI 51 – 73) CG: 77 (95% CI 63 – 91)
Mosti et al., 2011^(40)^	RCT	Unna’s boot (n = 50)	2 cohesive layers (n = 50)	12	Healing time^**^ IG: 49.5 (95% CI 27.7 – 69.7) CG: 48 (95% CI 33 – 63.5)
Lazareth et al., 2012^(41)^	RCT^♦^	2-layers (n = 93)	4-layers (n = 93)	12	**Healing rate p = .0165** IG: 44% (n = 41) CG: 39% (n = 36)
Weller et al., 2012^(42)^	RCT^♦^	3-layers (n = 23)	Short-stretch bandage (n = 22)	12	**Healing rate** *p* = .056 IG: 74% (n = 17) CG: 46% (n = 10)
Wong et al., 2012^(43)^	RCT	Short-stretch bandage (n = 107)	4-layers (n = 107) Usual care^ΔΔ^ (n = 107)	24	**Healing rate** IG: 72% (n = 77) CG1: 67.3% (n = 72) CG2: 29.0% (n = 31) Healing time^*^ *p* < .001 IG: 9.8 (± .77) CG1: 10.4 (±.80) CG2: 18.3 (±.86) **VU area difference** ^**###**^ IG: 2.85 (±8.18) *p* = .67 CG1: 3.39 (±8.64) *p* = .16 CG2: 6.90 (±10.62) ***p* = .047**
Luz et al., 2013^(44)^	Cohort	Unna’s Boot (n = 32)	Usual care (n = 11)	12	**UV area difference** ^#^ p > 0.05 IG: –47.12 (95% CI –100–107.41) CG: –53.06 (95% CI –100–57.96)
Macedo et al., 2013^(45)^	Cohort	Unna’s Boot (n = 18)	without comparator	10	**VU area difference** ^**#**^ **p = .000** IG: 73.5% (± 25.9)
Ashby et al., 2014^(46)^	ECR^♦♦♦^	2-layer elastic stockings (n = 230)	4-layers (n = 224)	53	**Healing rate** *p* = .96 IG: 71% (n = 163) CG: 70% (n = 157) Healing time^**^ *p = .96* IG:99 (95% CI 84–126) CG: 98 (95% CI 85–112)
Dolibog et al., 2014^(17)^	RCT	Unna’s Boot (n = 30)	Pneumatic compression^Δ^ (n = 28) Ulcer X Kit^ΔΔ^ (n = 30) Short-stretch multi-layer bandage^ΔΔΔ^ (n = 29) 2-layers stretch bandageΔΔΔΔ (n = 30)	NI	**Healing rate p = .03** IG: 20% (n = 6) CG1: 57.14% (n = 16) CG2: 56.66% (n = 17) CG3: 58.62% (n = 17) CG4: 16.66% (n = 5) **VU area difference** ^**###**^ IG: 15.78 (±19.57) p = .03 CG1: 10.13 (±20.88) p = .01 CG2: 9.67 (±20.02) p = .01 CG3: 8.12 (±17.23) p = .01 CG4: 16.27 (±20.23) p = .03
Finlayson et al., 2014^(47)^	RCT	4-layers (n = 53)	Class III elastic stockings (n = 50)	24	**Healing rate** *p* = .14 IG: 84% (n = 45) CG: 72% (n = 36) Healing time^*^ *p* = .03 IG:10 CG: 15 **UV area difference** ^#^ *p* = .27 IG: 96% (±15.6) CG: 93% (±14.9)
Lullove and Newton, 2014^(48)^	Retrospective cohort	Unna’s Boot (n = 60)	without comparator	12	**VU area difference^##^ p < .001** IG: 63.3%
Salome et al., 2014^(49)^	Cohort	Unna’s Boot (n = 50)	without comparator	53	**Healing rate p = .0001** IG: 84% (n = 42)
Abreu and Oliveira, 2015^(10)^	RCT	High compression elastic bandage (n = 9)	Unna’s Boot (n = 9)	13	**VU area difference## p < .0001** IG:42,32% CG: 69.41%
Guest et al., 2015^(50)^	Retrospective cohort	2 cohesive layers (250)	2-layers^Δ^ (n = 250) 4-layers^ΔΔ^ (n = 175)	24	**Healing rate** IG: 51% (n = 128) – IG x CG1 ***p* = .03** CG1: 40% (n = 100) – CG1 x CG2 ***p* = .05** CG2: 28% (n = 49) – IG x CG2 ***p* = .001** Healing time^***^ IG: 2.5 (±0.2) CG1: 2.4 (±0.2) CG2: 2.5 (±0.3) **UV area difference#** IG: 60% CG1: 58% CG2: 57%
Januário et al., 2015^(51)^	RCT	Unna’s Boot (n = 30)	90% trichloroacetic acid^Δ^ (n = 30) 20% carboxymethylcellulose^ΔΔ^ (n = 30)	20	**Healing rate** IG: 43% (n = 13) CG1: 13.3% (n = 4) CG2: 6.7% (n = 2) **VU area difference** ^**#**^ **p < .022** IG: 66.9 (±6.5) CG1: 46.4 (±5.8) CG2: 44.7 (±5.0)
Coutinho, 2019^(52)^	Cohort	High compression elastic bandage (n = 48)	without comparator	04	**VU area difference** ^**##**^ **p < .001** IG: 72.9%
Gillet et al., 2019^(53)^	RCT^♦^	2-layers (n = 47)	4-layers (n = 41)	16	**Healing rate p < .001** IG 48.9% (n = 23) CG: 26.3% (n = 11) **Healing time p = .03** IG: OR 3.01 (97.5% CI 1.1–8.6)
Folguera-Álvarez et al., 2020^(54)^	RCT^♦^	2-layers (n = 56)	Usual care (n = 37)	12	**Healing rate** IG: 57.1% (n = 32) CG: 67.5% (n = 25) Healing time^**^ p *= .744* IG: 45 CG: 60
Mosti et al., 2020^(55)^	RCT^♦^	Inelastic stockings (n = 33)	2-layers (n = 33)	12	**Healing rate** IG: 78.7% (n = 26) GC: 69.6% (n = 23)
Stather et al., 2021^(56)^	RCT	Inelastic stockings (n = 20)	2-layers (n = 20)	26	**Healing rate** IG: 60% (n = 12) CG: 55% (n = 11) Healing time^*^ IG: 12.67 (±6.11) CG: 13.64 (±6.98)
Senet et al., 2022^(57)^	Multicenter cohort	Single-layer multicomponent bandage (n = 52)	without comparator	6	**Healing rate** IG: 18 (35%) Healing time^**^ IG: 33 (±12)
Souza et al. 2022^(58)^	Cohort	Unna’s Boot (n = 14)	without comparator	9	**UV area difference** ^**###**^ p = 1.00 IG: 9.33 (±7.81)
Karanikolic et al., 2023^(59)^	RCT	Class III compression stockings (n = 56)	Elastic bandage + Class III compression stockings (n = 60)	24	**Healing rate** CG: 55% (n = 33)
Ulusoy and Iscan, 2023^(60)^	Cohort	4-layers (n = 113)	without comparator	12	**Healing rate** IG: 30 (26.5%) Healing time^*^ IG: 23.2 (±13.8)

**Source:** Prepared by the authors, 2024.

^♦^multicenter RCT;

^♦♦^multicenter cross-over RCT;

^♦♦♦^multicenter pragmatic RCT;

*weeks;

**days;

***months;

^#^% reduction;

^##^(cm^2^/week);

^###^ ≠ initial-final VU area;

^Δ^CG1;

^ΔΔ^CG2;

^ΔΔΔ^CG3;

^ΔΔΔΔ^CG4.

Digital planimetry was the most used outcome measurement method (n = 15; 38.7%). In the study results, 18 (46.2%) reported that no statistically significant difference was found (*p* < .05) among the CTs analyzed regarding healing. Of the 21 (53.8%) studies that found differences, four (10.2%) were cohort studies, without comparators, three (7.7%) with UB and one (2.6%) with high compression elastic bandage. Of the RCTs, 16 (41%) found differences related to the healing rate (n = 11; 28.2%), the VU area (n = 9; 23.1%), and the time to healing (n = 7; 17.9%).

Two RCTs that had UB as one of the CTs found no differences regarding VU healing. One compared it to 4-layer bandage^(31)^ and the other with a 2-layer cohesive bandage^(40)^. In a study that compared the Ulcer X system and the multilayer short-stretch bandage, the results showed that these two systems were superior in healing rate and the difference between the initial and final VU areas compared to UB and the 2-layer short-stretch bandage^(17)^. In contrast, in two other RCTs^(10,51)^, UB was superior in terms of the difference between the initial and final area. In the first, the effect of UB was compared to usual care with carboxymethyl cellulose and trichloroacetic acid^(51)^ and in the other compared to high-compression elastic bandage^(10)^.

In the cohort studies (n = 3) evaluating UB, considering only the measure of effect between the initial and final assessment, two identified a significant reduction in the VU area at the end of follow-up^(45,48)^ and another identified a more significant healing rate^(49)^.

Some studies included^(26,31,34,37,39,42,43,50)^ analyzed independent factors associated with healing and identified some evidence. The first was the duration of the UV with healing time^(26,50)^, that is, more recent UV healed faster. The duration of the VU was also directly related to the healing rate^(42,50)^. Others found that the size of the initial VU influenced healing time, smaller VUs healed faster^(31,34,37,39,43)^. One found a relationship between the initial size of the VU and the rate of healing, with smaller VUs healing at a greater rate^(42)^.

### Adverse Events

Of the studies included, 17 reported occurrences of AE, ten of which were related to 4-layer bandages; six with short-stretch bandages; three with UB and 2-layer bandage; two with 2-layer cohesive and 3-layer bandages. The four-layer bandage was the most used among studies reporting the events and had the highest number of adverse events reported. As for the total, the most cited adverse event was the appearance of a new ulcer, with 73 reports.

### Risk of Bias

**Figure 2:**
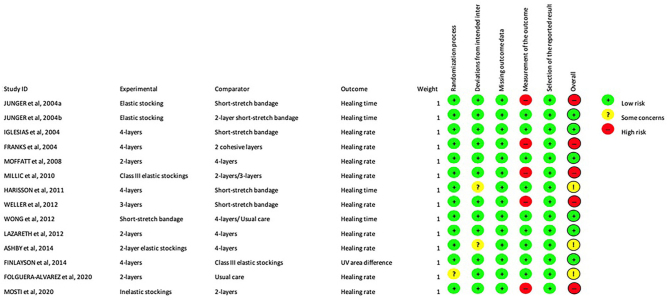
Assessment of risk of bias of RCT with intention-to-treat analysis.

**Source:** Prepared by the authors in the ROB-2 tool, 2022.

Of the 39 studies, 28 (71.8%) were RCTs. The RCTs were evaluated with the ROB-2 tool^(20)^, regarding the objective of statistical analysis, of evaluation of the effect of attribution to the intervention, intention-to-treat effect (ITT), and effect per protocol (PP). The tool separates the evaluation according to the type of effect under study. Figure 2 shows the 14 (50%) RCTs included in the study that were performed with ITT. According to the analysis, six (42.9%) studies were considered at low risk of bias, three (21.4%) with some concerns, and five (35.7%) with high risk of bias. Of the 14 (50%) RCTs that performed PP analysis, one (7.1%) was assessed with low risk of bias and 13 (92.9%) with high risk of bias. Cohort and case-control studies were evaluated with the ROBINS-I tool^(21)^. In this assessment, the ten (25.6%) cohort studies and the only (2.5%) case-control study presented a serious risk of bias.

### Summary of Results

**Figure 3:**
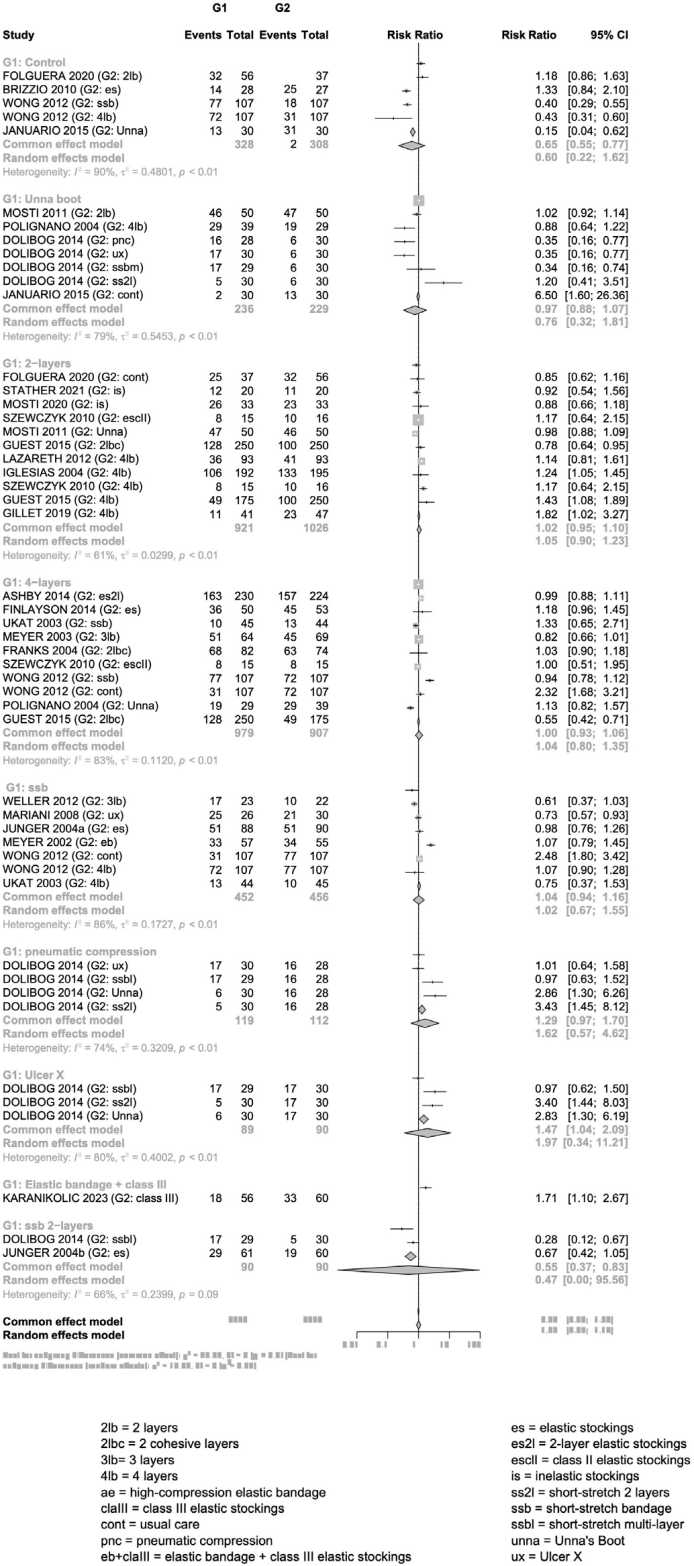
Meta-analysis of the number of healed ulcers compared among compression therapy groups.

**Source:** Prepared by the authors, 2024.

Of the 39 studies included in the SR, only 25 (64.1%) could undergo meta-analysis. As mentioned in the methods, the meta-analysis was organized into subsets, according to the results presented. In the UV healing rate subset, the forest plot (Figure 3) shows the number of healed ulcers in the 25 included studies, organized into CT groups, compared one by one. Of these, four (10.3%) used UB and this proved to be superior for treatment in only one study^(51)^. This study compared^(51)^ UB (n = 30) with usual care (n = 30), demonstrating a 6.5 greater chance of healing with the use of UB (RR 6.50 – 95% CI 1.6 – 26.36)^(51)^. In comparison with other CTs, in terms of the number of ulcers healed during follow-up, UB proved to be equivalent in the study that compared it with 2-layer bandages^(40)^ (RR 1.02 – 95% CI 0.92 – 1.14), in one comparing it with a 4-layer bandage^(31)^ (RR 0.88 CI 95% 0.64 – 1.22) and in one comparing it with a 2-layer short-stretch bandage^(17)^ (RR 1.20 CI 95% 0.41 – 3.51). In the study that compared UB with other CTs^(17)^, UB was lower in the number of UV healed when compared with pneumatic compression (RR 0.35 CI 95% 0.16 – 0.77), with Ulcer X (RR 0.35 CI 95% 0.16 – 0.77), and with multilayer short-stretch bandage (RR 0.34 CI 95% 0.16 – 0.74).

In the time to VU healing subset, 16 studies were included. Among the three who used BU, no superior effect of this CT on VU healing time was identified. In the VU area difference subset, five studies were included. Of these, four used UB and this proved to be superior for treatment in only one study. This studycompared UB (n = 9) with high compression elastic bandage (n = 9), resulting in a significant mean reduction in the VU area (–23.62, 95% CI –41.07 – –6.17)^(10)^. Among the other CT and usual care, UB proved to be equivalent for reducing the VU area throughout the follow-up.

In this SR, the assessment of heterogeneity was limited. Only five studies compared the same compression therapies, 2-layer bandage versus 4-layer bandage (Figure 3). Regarding the assessment of publication bias, even though the searches reached a good scope with the inclusion of 25 studies in the meta-analysis, few shared the same interventions and the intervention of interest in this SR, which resulted in a small sample, with less than ten studies. Therefore, it was not possible to assess publication bias.

## DISCUSSION

Despite the predominance of studies on the European continent, investigations were found in several countries around the world, reinforcing that VU is a public health problem^(2,3)^. Furthermore, the results reinforce the higher incidence of CVI and VU in the older people and women^(1,61,63)^. Two studies^(39,50)^ found an association between age and time for VU healing, showing that older people had their ulcers healed in a longer time than younger people.

Among the CT used, the 4-layer bandage was the most used, followed by the UB, the 2-layer bandage, and the short-stretch bandage. The finding reinforces multilayer therapies as a frequent option, and secondly, inelastic therapies such as UB and short stretch bandage^(5,6)^.

Regarding the healing outcome, of the total number of studies that used UB in one of its groups, it was observed that the majority were carried out in Brazil, reinforcing the argument that it is a CT commonly used in the country^(45,49)^ and, at the same time, a concern on the part of Brazilian researchers to seek evidence to support this clinical practice. On the other hand, only three were RCTs. Considering the relevance of this type of design for evaluating the effect of an intervention^(64)^, this finding reinforces the importance of carrying out RCTs evaluating the effect of UB in patients with venous ulcers, promoting the best evidence.

Still regarding healing, it was observed that there was no uniformity in the presentation of results and different measurements of the outcome were used, hindering the performance of a meta-analysis covering all studies. It is suggested that studies that evaluate this outcome be carried out based on the initial area and final area of the VU, the time for healing, and the percentage of VU healed at the end of the follow-up.

As for the method of verifying healing characteristics, digital planimetry and photography were the most frequent forms of measurement. Although the calculation of the VU area in digital planimetry is computerized, the UV tracing in most studies was carried out manually, a method considered a reference in the literature^(65)^.

In the meta-analysis, UB was superior for healing compared to usual care in one study^(51)^ and, in another, in relation to the high compression elastic bandage^(10)^. Contrasting the findings of the present investigation, a SR^(12)^ identified, with a moderate degree of evidence, an indifference in VU healing rates when comparing UB with other CTs.

The results of the studies found that compared CT with usual care for the healing outcome reinforced the findings described in two meta-analyses^(5,6)^, which mention that using some type of compression is superior to not using it. When comparing different types of CT, it was observed that the findings are confirmed, that is, multilayer bandages are more effective; 4-layer bandages were superior to short-stretch bandages, but equivalent to 2-layer bandages, and elastic stockings were superior to short-stretch bandages^(17,28,30,32,34,37,43)^.

Although the studies included identified superiority in their analyses, there is probably no significant difference between the CTs; however, there is evidence of its use as superior to usual care. The justification is possibly due to the therapeutic effect being also related to the characteristics of VU, self-care, adherence and access to treatment, and tolerance to the CT used.

This SR did not evaluate the effect of all therapies. In spite of this, it should be noted that although other CTs with more technology are efficiently superior to UB, this latter has been used due to low cost, although there is no economic analysis to support this statement. Thus, it is a treatment option for countries with limited health resources^(12)^.

An integrative review of the literature that analyzed studies regarding the types of CT in VU, emphasizing the use of UB, found broad support for the use of UB due to its effective curative action and lower costs. In addition, eight studies included in the review indicated the positive effect of UB in controlling edema, reducing the area, and healing injuries, as well as improving the individuals’ quality of life^(66)^. On the other hand, UB may require longer healing time compared to multilayer bandaging, as its mechanism of action depends on ambulation^(11,67)^.

In addition to the evidence regarding the effectiveness of CT, it should be noted that the choice of therapy needs to consider the severity of the CVI^(3)^, the size and duration of the VU, calf circumference, and ankle mobility. Another important fact to be considered is to which extent the patient adapts to the therapy used and demonstrates better adherence. Some studies evaluated patient comfort and/or satisfaction in using the therapy^(31,32,35,37,42,55)^; for instance, a study is cited that identified that comfort, pain when applying therapy, and ease to put shoes on improved throughout the treatment; however, no difference was found between the CTs used^(25)^.

Therefore, considering the prevalence of VU, the diversity and lack of robust results in the evidence, the performance of intervention studies comparing different types of CT for the treatment of VU with cost-effectiveness analysis and outcome assessments, considering the initial and final moment of follow-up is suggested.

Observing the AEs reported in studies, it should be highlighted that pain appears to be a very frequent event, although the expected outcome with the use of CT is its reduction. Considering the type of intervention, it was observed that UB was present in 10% of reported events and 4-layer CT, as it is proportionally the most used, presented a greater number of reports. Considering the severity and relationship of AEs with the intervention, most of the reported events can be considered mild and moderate, but also associated with the clinical signs of CVI and VU, such as pain, skin maceration, and the opening of new ulcerations.

Analyzing the quality of evidence of the studies included, the majority were from RCTs, but with a high risk of bias. This result was influenced by the lack of information about blinding in the evaluation and analysis of results. Most studies reported that, due to the nature of the treatment, it was not possible to blind the patients and professionals who applied the intervention. However, few mentioned whether there was blinding of the outcome evaluator and/or of the professional who analyzed the results. Furthermore, in some investigations, the outcome evaluator was the same professional who applied the intervention, a situation that compromises the degree of evidence in the study, increasing the risk of bias in assessing the effect of the intervention^(20)^. Considering the evaluations carried out in the research included in this SR, it is important to highlight that the study that found superiority of UB over high-compression elastic bandages for VU healing^(10)^ was considered to have a high risk of bias due to the lack of information about blinding in the outcome assessment.

The high degree of risk of bias in the included studies, the short follow-up period, the limited number of patients, the diversity of therapies and outcome assessments affected the analysis of results and the robustness of the evidence. These findings are consistent with the 2012 SR, in which most RCTs had small samples and uncertain or high risk of bias.^(5)^.

In the light of this, and considering the homogeneity in the quality of evidence among the studies, it was concluded that the results they found, using the GRADE system, presented a moderate degree of evidence. However, it is understood that the evidence for estimating the effect of CT on healing may be modified by future studies.

## LIMITATIONS

The limitations were related to: diversity in the presentation of results by the included studies, a fact that did not allow meta-analysis of all studies and heterogeneity assessment to be carried out; inclusion of studies published only in English, Portuguese and Spanish; limitation of access to some studies in full for free.

## CONCLUSION

Although UB is still quite frequently used, no evidence was found that it is more effective for UV healing when compared to other CTs. However, it appears to be effective when compared to usual care. Thus, given the scenario of provision of bandages without compression classification, as occurs in Brazil, UB still appears to be the best therapeutic option for treating users with VU.

Considering the variety of CTs on the market, the selection of CT according to the severity of CVI, and the few studies with statistical differences among CTs, it is highlighted that carrying out RCTs with cost-effectiveness analysis can contribute to therapeutic choice. Given the context of widespread use of UB in Brazil, more studies still need to be carried out to evaluate its real effectiveness.

Regarding the quality of the evidence, carrying out some method of blinding in evaluating the effect of therapies, in analyzing the results, is essential, as well as clear mention of the form of blinding adopted. Failure to comply with this requirement compromises the quality of the study, impacting the reduction of scores in the evidence quality analysis.

The results of this SR can contribute to the performance of health professionals by presenting scientific evidence that reinforces the superiority of the use of any CT in relation to usual care, contributing to decision-making and providing support for discussing the supply of inputs in health services.
