# Supplementary material for: Effect of Unna's boot on venous ulcer healing: a systematic review and meta-analysis
Source: Rev Esc Enferm USP. 2024 Sep 2;58:e20230397. doi: 10.1590/1980-220X-REEUSP-2023-0397en (PMC11368076; doi:10.1590/1980-220X-REEUSP-2023-0397en)
Supplement: Translation

# Translation: Efeito da Bota de Unna na cicatrização de úlceras venosas: revisão sistemática e metanálise

**DOI:** 10.1590/1980-220X-REEUSP-2023-0397pt

**Authors:** Fernanda Peixoto Cordova, Ana Claudia Furhmann, Andreia Cristina Feitosa do Carmo, Eduardo Nunes Vales, Diego Henrique Terra, Bárbara Uuritz da Silva, Diani de Oliveira Machado, Amália de Fátima Lucena, Lisiane Manganelli Girardi Paskulin

**Affiliations:** 1 Universidade Federal do Rio Grande do Sul, Escola de Enfermagem, Porto Alegre, RS, Brasil.; 2 Universidade Federal de São Paulo, Biblioteca, São Paulo, SP, Brasil.; 3 Universidade Federal de Ciências da Saúde, Faculdade de Medicina, Porto Alegre, RS, Brasil.; 4 Centro Universitário Ritter dos Reis, Escola de Enfermagem, Porto Alegre, RS, Brasil.; 5 Hospital Nossa Senhora da Conceição, Porto Alegre, RS, Brasil.

**Keywords:** Úlcera Varicosa, Bandagens Compressivas, Meias de Compressão, Revisão Sistemática, Metanálise

## Abstract

**Objetivo:**

Analisar o efeito da Bota de Unna na cicatrização de úlceras venosas em comparação com outras terapias.

**Métodos:**

Revisão Sistemática realizada nas bases de dados *Scopus*, *Embase*, *Cochrane Library*, *Web of Science*, *PubMed*, *Cumulative Index of Nursing and Allied Health Literature*, Literatura Latino-Americana e do Caribe em Ciências da Saúde, e de literatura cinzenta. População- pacientes adultos com úlcera venosa; Intervenção- Bota de Unna (BU); Controle- outras terapias compressivas (TC); Desfecho- cicatrização; Delineamentos- ensaio clínico randomizado, estudo de coorte e caso controle, publicados de 2002 a 2023. Avaliaramu-se efeito da intervenção, risco de viés e qualidade da evidência. Registrada no PROSPERO (CRD42021290077).

**Resultados:**

Foram incluídos 39 estudos, com 5.151 pacientes. A maioria (71,8%) era ensaios clínicos randomizados (ECR). A BU foi utilizada como intervenção/controle em oito estudos. Na comparação entre TC, somente 1 estudo com BU apresentou efeito superior (p < .001) na cicatrização, comparado com atadura elástica de alta compressão. Na análise de qualidade da evidência, 27 estudos foram avaliados com alto risco de viés.

**Conclusão:**

Não foi encontrada superioridade da BU na cicatrização de úlceras venosas quando comparada com outras TC.

## INTRODUÇÃO

A úlcera venosa (UV) é um desfecho grave da Insuficiência Venosa Crônica (IVC)^(1)^, representando 85% dos casos de úlceras crônicas de perna com prevalência entre 1,5 e 3% na população^(2,3)^. O padrão ouro no tratamento da UV é a aplicação de terapia compressiva (TC)^(1,3)^, que consiste na aplicação de compressão externa à perna para favorecer o retorno venoso e a reabsorção do edema, reduzir a hipertensão e a estase venosa, contribuindo para a cicatrização e diminuindo a recorrência de UV^(1,4)^.

A TC pode ser estática ou dinâmica. A terapia estática é produzida por ação elástica ou inelástica de ataduras/bandagens e meias de compressão. A terapia dinâmica é realizada por compressão pneumática intermitente^(1,2)^. Dentre as TC estáticas, existem diversos tipos, classificadas de acordo com tipo e número de componentes e/ou camadas utilizados, efeito e níveis de compressão aplicados^(1,2)^.

Neste contexto, Revisões Sistemáticas (RS) foram conduzidas abordando diferentes TCs. Em um estudo de 2012 com 59 ensaios clínicos randomizados (ECR) (n = 4.321) concluiu-se que a TC aumenta as taxas de cicatrização de UV em comparação ao uso de bandagens sem graduação de compressão, ou seja, aquelas de tecidos como crepe, Rayon^®^ ou de tecido sintético misto. Além disso, foi identificado que os sistemas de compressão com multicomponentes e com multicamadas, especialmente os com bandagem elástica, foram mais eficazes que os sistemas com um único componente ou compostos principalmente por bandagens inelásticas. Não foram encontradas evidências significativas para cicatrização entre as demais terapias, como, por exemplo, a Bota de Unna (BU), comparada com bandagens de quatro camadas, meias elásticas e meias inelásticas ajustáveis^(5)^.

Outra RS envolvendo 14 ECR (n = 1.391 pacientes) avaliou o efeito das bandagens de curto estiramento, bandagens de quatro camadas e BU comparadas à ausência de compressão. Os resultados demonstraram celeridade na cicatrização no uso de qualquer bandagem em comparação à ausência de compressão e indicaram possível melhora em alguns aspectos da qualidade de vida e da dor^(6)^.

Os resultados das RS citadas indicam que o uso de TC é mais efetivo para cicatrização do que a sua não utilização. Contudo, devido à existência de diversos tipos de TC, não existe consenso mundial quanto ao uso de um tipo específico^(1,2,5,7)^. No Reino Unido, a bandagem de quatro camadas é amplamente aplicada, enquanto na Europa continental e na Austrália a bandagem de curto estiramento é uma prática mais frequente. Já no Brasil e nos Estados Unidos, a BU é a mais comum^(5,8)^. Contudo, no Brasil, para o tratamento de pessoas com UV ainda se observa um amplo uso de bandagens sem classificação de compressão^(9)^, ou seja, bandagens de tecidos comuns.

A BU, inventada em 1885, consiste em uma bandagem de compressão inelástica com ação a partir do aumento da pressão venosa durante a contração muscular, principalmente na deambulação^(10,11)^. Uma RS realizada com 08 estudos (n = 643 pacientes) teve como objetivo determinar a efetividade da BU no tratamento de UV, comparando a BU aos outros tipos de TC, associadas ou não a coberturas primárias. Na metanálise, quanto à taxa de cicatrização não foi encontrada diferença entre as terapias (OR 0,45), e quanto ao tempo de cicatrização a BU teve cicatrização mais lenta^(12)^.

Como já mencionado, embora a BU tenha sido inventada há bastante tempo e mesmo diante de diversos tipos de terapias compressivas, ela ainda é bastante utilizada no tratamento da UV no contexto mundial, principalmente no Brasil, nos serviços de Atenção Primária à Saúde. Neste contexto, apesar de a observação clínica empírica demonstrar aparentes efeitos satisfatórios na cicatrização, boa parte das evidências científicas que compararam a BU com outras terapias não foram robustas o suficiente para identificar e justificar sua ampla utilização e medir se o efeito percebido na prática clínica é estatisticamente significativo. Além disso, estes estudos não são recentes. Diante disso, acredita-se que uma nova avaliação de estudos referentes ao uso da BU pode contribuir para o aprimoramento do manejo clínico e auxiliar na formulação de diretrizes e estratégias de cuidado mais assertivas, bem como na implementação de insumos efetivos para o cuidado ao paciente, principalmente no Brasil, nos serviços de Atenção Primária à Saúde, porta de entrada e principal local de atendimento aos usuários com UV. Portanto, este estudo teve como questão de pesquisa: Qual é o efeito da Bota de Unna na cicatrização de úlceras venosas comparado com outras terapias compressivas?

## MÉTODOS

### Protocolo e Registro

Trata-se de uma Revisão Sistemática com Metanálise elaborada segundo as recomendações da *Cochrane* ^(13)^, apresentada de acordo com o *Preferred Reporting Items for Systematic Reviews and Meta-Analyses* (PRISMA)^(14,15)^. O protocolo do estudo foi registrado no PROSPERO (CRD42021290077), com o título: *Effect of Unna Boot on healing, pain, edema and quality of life in patients with venous ulcers: Systematic Review* ^(16)^.

### Critérios de Elegibilidade

O mnemônico PICOS foi: População- pacientes adultos com úlcera venosa; Intervenção- Bota de Unna (BU); Controle- outras terapias compressivas (TC); Desfecho- cicatrização; Delineamentos- ensaio clínico randomizado, estudo de coorte e caso controle. A intervenção de interesse foi a TC do tipo BU. O comparador foram as outras TC^(1,2)^. Foram incluídos estudos realizados com adultos, com UVs em tratamento com TC, com comparações entre diferentes terapias e TC com bandagens sem classificação de compressão, denominada como cuidado usual. Foram incluídos estudos publicados no período de novembro de 2001 a janeiro de 2024, disponíveis na íntegra, na língua portuguesa, inglesa e espanhola. Quanto ao delineamento, foram inseridos ECR para avaliar os efeitos benéficos do tratamento, complementados por resultados de estudos observacionais (estudos de coorte e caso-controle). Os estudos incluídos apresentaram cicatrização tanto como desfecho primário quanto secundário. Estudos observacionais que apresentaram apenas um grupo de intervenção foram incluídos, desde que essa intervenção fosse a BU.

Foram excluídos estudos que compararam o efeito de TC pneumática com outras terapias, a exceção de um deles que trouxe a BU como uma das TC de intervenção^(17)^. Não foram incluídos estudos com cointervenções associadas às TC, tais como procedimentos cirúrgicos, invasivos, tratamentos farmacológicos e curativos, para reduzir a interferência no efeito dos desfechos analisados.

### Fontes de Informação

As buscas foram realizadas em janeiro de 2024, nas bases de dados: *Scopus, Embase, Cochrane Library, Web of Science, PubMed, Cumulative Index of Nursing and Allied Health Literature (CINAHL Complete)*, Literatura Latino-Americana e do Caribe em Ciências da Saúde (LILACS), como também nas bases de dados de literatura cinzenta – *Grey literature* (opengrey.org) e no Banco de Teses e Dissertações da Coordenação de Aperfeiçoamento de Pessoal de Nível Superior (CAPES).

### Extração de Dados

A elaboração das estratégias de buscas por bases de dados, as buscas nas bases de dados e o registro dos achados no *software Rayyan Intelligent Systematic Review* ^(18)^ foram realizados por bibliotecária experiente em RS. A busca na literatura no Banco de Teses e Dissertações e *Open Grey* foi realizada por dois pesquisadores de forma individual, utilizando termos de buscas simples.

A seleção dos estudos foi realizada por dois pesquisadores de forma independente. Utilizou-se o *software Rayyan* para identificar possíveis duplicações. Após, foi realizada a leitura dos títulos e resumos, os pesquisadores classificaram os estudos individualmente e a ferramenta analisou a concordância e os conflitos entre as seleções. Houve concordância em 32 estudos, 17 foram conflitantes e cinco foram classificados como talvez. Os conflitos foram resolvidos por meio de discussão entre os pesquisadores ou com a intervenção de um terceiro. Após a seleção final, os estudos foram avaliados na íntegra de forma independente.

### Avaliação da Qualidade do Estudo

Foi utilizado o Sistema *Grading of Recommendations Assessment*, *Development and Evaluation* (GRADE) para avaliar a qualidade do corpo de evidências do desfecho em análise nos estudos incluídos. O Sistema GRADE define a certeza de um corpo de evidências com relação à extensão em que se pode ter certeza de que uma estimativa de efeito ou associação está próxima da quantidade real de interesse específico. A avaliação da certeza de um corpo de evidências usando o GRADE envolve a consideração do risco de viés dentro do estudo (qualidade metodológica), objetividade da evidência, heterogeneidade, precisão das estimativas de efeito e risco de viés de publicação^(19)^.

Resultados de desfechos provenientes de ECR recebem inicialmente pontuação de alta qualidade, enquanto aqueles gerados por estudos observacionais começam como baixa qualidade. Na sequência, aplica-se o sistema de ponderação para diminuir ou aumentar a pontuação da qualidade da evidência. A qualidade da evidência é classificada em quatro níveis: alta, moderada, baixa ou muito baixa^(13,19)^.

### Análise Estatística

As informações coletadas dos estudos incluídos foram: autores, título, ano de publicação, periódico/fonte publicação, país de origem; objetivos; tipo de estudo; população em estudo; critérios de inclusão e exclusão dos participantes; amostra; descrição da amostra; local do estudo; recrutamento; randomização; cegamento; intervenção; controle; tempo de seguimento; desfechos; outros desfechos reportados; resultados; limitações; medidas de efeito; efeito de interesse; eventos adversos; interpretações de resultados; conclusões. Os resultados foram organizados no programa editor de planilhas Excel.

Na análise do desfecho cicatrização foram considerados os resultados apresentados quanto ao tempo (semanas) para cicatrização da UV (média e desvio padrão); taxa de cicatrização de UV (n e %) durante o seguimento e diferenças da área (cm^2^) da UV inicial e final (média e desvio padrão). Na avaliação deste desfecho, esperava-se que as UV cicatrizassem no menor tempo possível, que o percentual de UV cicatrizadas fosse maior ou que ocorresse uma redução significativa da área da UV inicial para a final sob efeito da BU, comparada com as outras TC. Foi realizada a organização dos dados em subconjuntos: taxa de cicatrização, tempo para cicatrização e diferenças da área da UV.

### Risco de Viés

Na avaliação do risco de viés para os ECR foi utilizada a ferramenta *Cochrane Risk of Bias* (ROB-2 *tool* )^(20)^. Os estudos de coorte e caso-controle foram avaliados a partir da ferramenta *Risk Of Bias In Non-randomized Studies – of Interventions* (ROBINS-I *tool* )^(21)^.

Com relação à avaliação do viés de publicação, não se encontrou o número de estudos suficientes para realização. Foi utilizado o *Sistema Grading of Recommendations Assessment, Development and Evaluation* (GRADE) para avaliar a qualidade do corpo de evidências para o desfecho^(22)^.

### Metanálise

As estimativas conjuntas de efeito da intervenção foram realizadas utilizando o *software RStudio*. A metanálise do desfecho cicatrização foi realizada em subconjuntos, conforme o resultado apresentado nos estudos, descrito em análise estatística. Para os estudos que apresentaram as medidas em mediana, intervalo interquartílico, mínimo e máximo, realizou-se a conversão para média e desvio padrão, segundo as fórmulas de conversão^(23)^.

### Síntese dos Resultados

Na análise descritiva, os dados dos estudos incluídos foram expostos em um quadro, considerando os seguintes itens: autor e ano, país e idioma, periódico/fonte, objetivo principal, desenho do estudo, amostra total. Outro quadro apresentou dados de intervenção, controle, desfechos analisados, medida do desfecho, tempo de seguimento e principais resultados. Conforme orientação do *check list* do PRISMA, foram ainda apresentados os Eventos Adversos (EA) relatados nos estudos incluídos.

Para a análise quantitativa, os estudos precisavam ser homogêneos em termos de população, exposição, comparador e resultado. No desfecho cicatrização foram agrupados os estudos semelhantes quanto à existência de comparação entre TC, conforme os subconjuntos identificados neste desfecho. A metanálise foi realizada com o gráfico *forest plot* em cada subconjunto para avaliação da eficácia da intervenção BU na cicatrização de UV comparadas com outras terapias compressivas.

## RESULTADOS

### Seleção de Estudos

As buscas nas bases de dados resultaram em 5.048 estudos. Considerando os critérios definidos, foram incluídos 39 estudos, conforme apresentado no Fluxograma (Figura 1).

**Figura 1:** Fluxograma seleção de artigos de acordo com PRISMA 2020.

**Fonte**: Adaptado^(15)^ pelos autores, 2024.

Entre 39 estudos incluídos, a amostra total foi de 5.151 pacientes adultos com UV. Considerando os 28 estudos que estratificaram as características da amostra, a maioria era do sexo feminino (54,3%). Os estudos foram publicados entre os anos de 2002 e 2023, sendo mais frequente nos anos de 2004 e 2014 (n = 5; 12,8% respectivamente). Entre os anos 2010, 2012 e 2015 foram 3 estudos (7,6%) por ano respectivo; nos anos de 2003, 2008, 2011, 2013, 2019, 2020, 2022 e 2023 foram publicados 2 estudos (5,1%) e nos anos 2002, 2005, 2007 e 2021 foi publicado apenas 1 estudo (2,5%) por ano. Nos anos de 2006, 2009, 2016, 2017 e 2018, não foram publicados estudos. Com relação ao país de origem, o país que mais teve publicações foi o Reino Unido (n = 8; 20,5%), seguido do Brasil (n = 7; 17,9%), Itália (n = 4; 10,2%); contudo, somando aos demais estudos realizados em outros países, observa-se que majoritariamente (n = 25; 64,1%) os estudos eram de países europeus (Tabela 1).

**Tabela 1:** Síntese da caracterização dos estudos incluídos, Porto Alegre (RS), Brasil, 2022.

Variáveis	N	%
**Ano**		
2002	1	2,5
2003	2	5,1
2004	5	12,8
2005	1	2,5
2007	1	2,5
2008	2	5,1
2010	3	7,6
2011	2	5,1
2012	3	7,6
2013	2	5,1
2014	5	12,8
2015	3	7,6
2019	2	5,1
2020	2	5,1
2021	1	2,5
2022	2	5,1
2023	2	5,1
**País**		
Reino Unido	8	20,5
Brasil	7	17,9
Itália	4	10,2
Alemanha	3	7,6
Franga	3	7,6
Sérvia	3	7,6
Austrália	2	5,1
Estados Unidos da América	2	5,1
Polonia	2	5,1
Argentina	1	2,5
Canadá	1	2,5
China	1	2,5
Espanha	1	2,5
Turquía	1	2,5
**Tipos de estudo**		
ECR	14	35,8
ECR multicentrico	12	30,8
ECR multicentrico pragmático	1	2,6
ECR multicentrico cross-over	1	2,6
Coorte	8	20,5
Coorte retrospectiva	2	5,1
Caso-controle	1	2,6
**Sexo (28 estudos)**		
Feminino	2.247	54,3
Masculino	1.891	45,7
**Idade (média/dp) (28 estudos)**		
68,6 anos (± 6,0)		
**Intervengo**		
Bota de Unna	8	20-20,5
Bandagem de 4 camadas	7	17,9
Meia elástica	6	15,3
Bandagem de 2 camadas	5	12,8
Atadura de alta de compressao	3	7,7
Meia inelásticas	3	7,7
**Controle**		
Bandagem de 4 camadas	9	23,1
Bandagem de curto estiramento	9	23,1
Bandagem de 2 camadas	8	20,5
Atadura sem classificagao de compressao	5	12,8
Bota de Unna	2	5,1
**Tempo de Seguimento (Mediana/IQ)**		
Semanas	12	12-24

**Fonte:** Elaborado pelos autores, 2024.

Com relação ao tipo de estudo, 28 (71,8%) eram algum tipo de ECR. Quanto ao agrupamento das TC, a BU foi utilizada em 20,5% das investigações como TC de intervenção e em 5,1% como TC de controle. A mediana do tempo de seguimento nos estudos foi de 12 semanas^(12–24)^ (Tabela 1).

### Resultados dos Estudos

No Quadro 1 estão detalhadas as informações dos estudos incluídos. A forma de avaliação do desfecho cicatrização variou bastante entre os estudos e a maioria utilizou mais de uma. As avaliações foram: taxa de cicatrização (n = 30; 76,9%), tempo para cicatrização (n = 21; 53,8%) e diferença da área da UV (n = 16; 41,0%) expressos em percentual de redução, coeficiente de redução semanal e diferença de redução entre área inicial para final da UV.

**Quadro 1:** Síntese da caracterização dos estudos incluídos, Porto Alegre (RS), Brasil, 2022.

Autor ano	Tipo estudo	Grupo intervenção (GI) (n)	Grupo controle (GC) (n)	Follow-up (semanas)	Síntese dos principais resultados
Meyer et al., 2002^(24)^	Coorte	Atadura elástica de alta compressão (n = 57)	Bandagem de curto estiramento (n = 55)	26	**Taxa de cicatrização** *p* = .623 GI: 58% (n = 33) GC: 62% (n = 34) Tempo para cicatrização* GI: 10 (IC 95% 8–12) GC: 11 (IC 95% 9–13)
Meyer et al., 2003^(25)^	Coorte	3 camadas (n = 64)	4 camadas (n = 69)	56	**Taxa de cicatrização *p* = .031** GI: 80% (n = 51) GC: 65% (n = 45) Tempo para cicatrização* *p* = .040 GI: 12 (IC 95% 10 – 15) GC: 16 (IC 95% 13 – 21)
Ukat et al., 2003^(26)^	ECR	4 camadas (n = 44)	Bandagem de curto estiramento (n = 45)	12	**Taxa de cicatrização** GI: 30% (n = 13) GC: 22% (n = 10) Tempo para cicatrização* *p* = .03 OR 2.9 (IC 95% 1,1 – 7,5)
Franks et al., 2004^(27)^	ECR^♦^	4 camadas (n = 74)	2 camadas coesivas (n = 82)	24	**Taxa de cicatrização** *p* = .79 GI: 85% (n = 63) GC: 83% (n = 68)
Iglesias et al., 2004^(28)^	ECR^♦^	4 camadas (n = 195)	Bandagem de curto estiramento (n = 192)	24	**Taxa de cicatrização *p* = .005** GI: 68% (n = 133) GC: 55% (n = 106) Tempo para cicatrização** *p* = .12 GI: 92 (IC 95% 71–113) GC: 126 (IC 95% 95–157)
Junger et al., 2004a^(29)^	ECR^♦^	Meia elástica (n = 88)	Bandagem de curto estiramento (n = 90)	12	**Taxa de cicatrização** GI: 58% (n = 51) GC: 57% (n = 51) Tempo para cicatrização** *p* = .80 GI: 43 (±18.3) GC: 43,6 (±18.3) **Diferença área da UV#** GI: 67,6% GC: 59%
Junger et al., 2004b^(30)^	ECR^♦^	Meia elástica (n = 61)	Bandagem de curto estiramento 2 camadas (n = 60)	12	**Taxa de cicatrização *p* = .0129** GI: 47,5% (n = 29) GC: 31,7% (n = 19) Tempo para cicatrização** *p* = .0297 GI: 61 (±26) GC: 68 (±25)
Polignano et al., 2004^(31)^	ECR^♦^	4 camadas (n = 39)	Bota de Unna (n = 29)	24	**Taxa de cicatrização** *p* = .42 GI: 74% (n = 29) GC: 66% (n = 19) Tempo para cicatrização** *p* = .13 GI: 51 (IC 95% 7–175) GC: 49 (IC 95% 7–168) **Diferença área da UV** ^#^ *p* = .30 GI: 100% (IC 95% –283,3 – 100) GC: 100% (IC 95% –489,3 – 100)
Blecken et al., 2005^(32)^	ECR	Meia inelástica (n = 12)	4 camadas (n = 12)	12	**Taxa de cicatrização *p* = .0173** GI: HR 0.56 GC: HR 1 **Diferença área da UV** ^##^ ***p =* .0369** GI: 2.93 (±0.6) GC: 2.3 (±0.7)
Millic et al., 2007^(33)^	ECR	3 camadas com meia elástica e bandagem de médio estiramento (n = 75)	bandagem de médio estiramento com 2 camadas (n = 75)	52	Tempo para cicatrização** *p* < .001 GI: 133 (28-464) GC: 211 (61-438)
Mariani et al., 2008^(34)^	ECR^♦^	Ulcer X Kit (n = 26)	Bandagem de curto estiramento (n = 30)	16	**Taxa de cicatrização *p* = .011** GI: 96,2% (n = 25) GC: 70% (n = 21)
Moffatt et al., 2008^(35)^	ECR^♦♦^	2 camadas (n = 39)	4 camadas (n = 42)	08	**Taxa de cicatrização antes** ^♦♦^ *p* = .30 GI: 15,3% (n = 6) GC: 7,1% (n = 3) **Diferença área da UV após** ^♦♦#^ *p* = .88 GI: 27,8% GC: 42,2%
Brizzio et al., 2010^(36)^	ECR	Meia elástica (n = 28)	Cuidado usual (n = 27)	26	**Taxa de cicatrização** *p* = .210 GI: 50% (n = 14) GC: 67% (n = 18) Tempo para cicatrização** *p* = .942 GI: 68 (±40) GC: 69 (±39)
Millic et al., 2010^(37)^	ECR	Meia elástica Classe III (n = 42)	2 camadas^Δ^ (n = 46) 3 camadas^ΔΔ^ (n = 43)	26	**Taxa de cicatrização** GI: 25% – GI x GC2 ***p* = .000** GC1: 67% GC2: 74% – GC2 x GC1 ***p* = .0238** Tempo para cicatrização* *p* > .05 GI: 12 GC1: 11 GC2: 14
Szewczyk et al., 2010^(38)^	ECR	Meia elástica classe II (n = 15)	2 camadas^Δ^ (n = 16) 4 camadas^ΔΔ^ (n = 15)	12	**Taxa de cicatrização** *p* > .05 GI: 53,3% (n = 8) GC1: 62,5% (n = 10) GC2: 60% (n = 9) **Diferença área da UV** ^##^ ***p* < .001** GI: .44 GC1: .55 GC2: .63
Harisson et al., 2011^(39)^	ECR^♦^	4 camadas (n = 215)	Bandagem de curto estiramento (n = 209)	12	Tempo para cicatrização** *p* = .98 GI: 62 (IC 95% 51 – 73) GC: 77 (IC 95% 63 – 91)
Mosti et al., 2011^(40)^	ECR	Bota de Unna (n = 50)	2 camadas coesivas (n = 50)	12	Tempo para cicatrização** GI: 49.5 (IC 95% 27,7 – 69,7) GC: 48 (IC 95% 33 – 63,5)
Lazareth et al., 2012^(41)^	ECR^♦^	2 camadas (n = 93)	4 camadas (n = 93)	12	**Taxa de cicatrização *p* = .0165** GI: 44% (n = 41) GC: 39% (n = 36)
Weller et al., 2012^(42)^	ECR^♦^	3 camadas (n = 23)	Bandagem de curto estiramento (n = 22)	12	**Taxa de cicatrização** *p* = .056 GI: 74% (n = 17) GC: 46% (n = 10)
Wong et al., 2012^(43)^	ECR	Bandagem de curto estiramento (n = 107)	4 camadas^Δ^ (n = 107) Cuidado usual^ΔΔ^ (n = 107)	24	**Taxa de cicatrização** GI: 72% (n = 77) GC1: 67,3% (n = 72) GC2: 29,0% (n = 31) Tempo para cicatrização* *p* < .001 GI: 9.8 (± .77) GC1: 10.4 (± .80) GC2: 18.3 (± .86) **Diferença área da UV^###^** GI: 2.85 (±8.18) *p* = .67 GC1: 3.39 (±8.64) *p* = .16 GC2: 6.90 (±10.62) ***p* = .047**
Luz et al., 2013^(44)^	Coorte	Bota de Unna (n = 32)	Cuidado usual (n = 11)	12	**Diferença área da UV** ^#^ *p* > 0,05 GI: –47.12 (IC 95% –100 – 107,41) GC: –53.06 (IC 95% –100 – 57,96)
Macedo et al., 2013^(45)^	Coorte	Bota de Unna (n = 18)	sem comparador	10	**Diferença área da UV#** ρ **= .000** GI: 73,5% (± 25,9)
Ashby et al., 2014^(46)^	ECR^♦♦♦^	Meia elástica 2 camadas (n = 230)	4 camadas (n = 224)	53	**Taxa de cicatrização** *p* = .96 GI: 71% (n = 163) GC: 70% (n = 157) Tempo para cicatrização** *p* = .96 GI: 99 (IC 95% 84–126) GC: 98 (IC 95% 85–112)
Dolibog et al., 2014^(17)^	ECR	Bota de Unna (n = 30)	Compressão pneumática^Δ^ (n = 28) Ulcer X Kit^ΔΔ^ (n = 30) Bandagem multicamadas de curto estiramento^ΔΔΔ^ (n = 29) Bandagem de estiramento com 2 camadas^ΔΔΔΔ^ (n = 30)	NI	**Taxa de cicatrização *p* = .03** GI: 20% (n = 6) GC1: 57,14% (n = 16) GC2: 56,66% (n = 17) GC3: 58,62% (n = 17) GC4: 16,66% (n = 5) **Diferença área da UV** ^###^ GI: 15.78 (±19.57) p = .03 GC1: 10.13 (±20.88) p = .01 GC2: 9.67 (±20.02) p = .01 GC3: 8.12 (±17.23) p = .01 GC4: 16.27 (±20.23) p = .03
Finlayson et al., 2014^(47)^	ECR	4 camadas (n = 53)	Meias elásticas classe III (n = 50)	24	**Taxa de cicatrização** *p* = .14 GI: 84% (n = 45) GC: 72% (n = 36) Tempo para cicatrização* *p* = .03 GI: 10 GC: 15 **Diferença área da UV** ^#^ *p* = .27 GI: 96% (±15,6) GC: 93% (±14,9)
Lullove e Newton, 2014^(48)^	Coorte retrospectiva	Bota de Unna (n = 60)	sem comparador	12	**Diferença área da UV** ^#^ ***p* < .001** GI: 63,3%
Salome et al., 2014^(49)^	Coorte	Bota de Unna (n = 50)	sem comparador	53	**Taxa de cicatrização *p* = .0001** GI: 84% (n = 42)
Abreu e Oliveira, 2015^(10)^	ECR	Atadura elástica de alta compressão (n = 9)	Bota de Unna (n = 9)	13	**Diferença área da UV** ^#^ ***p* < .0001** GI: 42,32% GC: 69,41%
Guest et al., 2015^(50)^	Coorte retrospectiva	2 camadas coesivas (250)	2 camadas^Δ^ (n = 250) 4 camadas^ΔΔ^ (n = 175)	24	**Taxa de cicatrização** GI: 51% (n = 128) – GI x GC1 ***p* = .03** GC1: 40% (n = 100) – GC1 X GC2 ***p* = .05** GC2: 28% (n = 49) – GI x GC2 ***p* = .001** Tempo para cicatrização*** GI: 2.5 (±0.2) GC1: 2.4 (±0.2) GC2: 2.5 (±0.3) **Diferença área da UV#** GI: 60% GC1: 58% GC2: 57%
Januário et al., 2015^(51)^	ECR	Bota de Unna (n = 30)	ácido tricloroacético a 90%^Δ^ (n = 30) Carboximetilcelulose a 20%^ΔΔ^ (n = 30)	20	**Taxa de cicatrização** GI: 43% (n = 13) GC1: 13,3% (n = 4) GC2: 6,7% (n = 2) **Diferença área da UV# *p* = .022** GI: 66.9 (±6,5) GC1: 46.4 (±5,8) GC2: 44.7 (±5,0)
Coutinho, 2019^(52)^	Coorte	Atadura elástica de alta compressão (n = 48)	sem comparador	04	**Diferença área da UV# *p* < .001** GI: 72,9%
Gillet et al., 2019^(53)^	ECR^♦^	2 camadas (n = 47)	4 camadas (n = 41)	16	**Taxa de cicatrização *p* < .001** GI 48,9% (n = 23) GC: 26,3% (n = 11) **Tempo para cicatrização *p* = .03** GI: OR 3.01 (IC 97,5% 1,1–8,6)
Folguera-Álvarez et al., 2020^(54)^	ECR^♦^	2 camadas (n = 56)	Cuidado usual (n = 37)	12	**Taxa de cicatrização** GI: 57,1% (n = 32) GC: 67,5% (n = 25) Tempo para cicatrização** *p* = .744 GI: 45 GC: 60
Mosti et al., 2020^(55)^	ECR^♦^	Meia inelástica (n = 33)	2 camadas (n = 33)	12	**Taxa de cicatrização** GI: 78,7% (n = 26) GC: 69,6% (n = 23)
Stather et al., 2021^(56)^	ECR	Meia inelástica (n = 20)	2 camadas (n = 20)	26	**Taxa de cicatrização** GI: 60% (n = 12) GC: 55% (n = 11) Tempo para cicatrização* GI: 12.67 (±6.11) GC: 13.64 (±6.98)
Senet et al., 2022^(57)^	Coorte multicêntrica	Bandagem multicomponente de camada única (n = 52)	sem comparador	6	**Taxa de cicatrização** GI: 18 (35%) Tempo para cicatrização** GI: 33 (±12)
Souza et al. 2022^(58)^	Coorte	Bota de Unna (n = 14)	sem comparador	9	**Diferença área da UV** ^###^ *p* = 1.00 GI: 9.33 (±7.81)
Karanikolic et al., 2023^(59)^	ECR	Meia de compressão classe III (n = 56)	Bandagem elástica + Meia de compressão classe III (n = 60)	24	**Taxa de cicatrização** GC: 55% (n = 33)
Ulusoy e Iscan, 2023^(60)^	Coorte	4 camadas (n = 113)	sem comparador	12	**Taxa de cicatrização** GI: 30 (26,5%) Tempo para cicatrização* GI: 23.2 (±13.8)

^♦^ECR multicêntrico;

^♦♦^ECR cross-over multicêntrico;

^♦♦♦^ECR pragmático multicêntrico;

*semanas;

**dias;

***meses;

^#^% redução;

^##^(cm^2^/sem);

^###^ ≠ área UV inicial-final;

^Δ^GC1;

^ΔΔ^GC2;

^ΔΔΔ^GC3;

^ΔΔΔΔ^GC4.

**Fonte**: Elaborado pelos autores, 2024.

A planimetria digital foi o método de medida de desfecho mais utilizado (n = 15; 38,7%). Nos resultados dos estudos, 18 (46,2%) relataram que não foi encontrada nenhuma diferença estatisticamente significativa (*p* < .05) entre as TC em análise quanto à cicatrização. Dos 21 (53,8%) estudos que encontraram diferenças, quatro (10,2%) foram estudos de coorte, sem comparadores, sendo três (7,7%) com BU e um (2,6%) com atadura elástica de alta compressão. Dos ECR, 16 (41%) encontraram diferenças relacionadas à taxa de cicatrização (n = 11; 28,2%), na área da UV (n = 9; 23,1%) e no tempo para cicatrização (n = 7; 17,9%).

Dois ECR que tiveram BU como umas das TC não encontraram diferenças com relação à cicatrização de UV. Um comparou-a com bandagem de 4 camadas^(31)^ e o outro com bandagem de 2 camadas coesivas^(40)^. Em um estudo que comparou o sistema Ulcer X e a bandagem de curto estiramento multicamadas, os resultados mostraram que estes dois sistemas foram superiores na taxa de cicatrização e na diferença entre as áreas iniciais e finais da UV comparados à BU e à bandagem de 2 camadas com curto estiramento^(17)^. Em contrapartida, em outros dois ECR^(10,51)^, a BU foi superior quanto à diferença da área inicial e final. No primeiro, o efeito da BU era comparado ao cuidado usual com carboximetilcelulose e com ácido tricloroacético^(51)^ e no outro comparado à atadura elástica de alta compressão^(10)^.

Nos estudos de coorte (n = 3) que avaliaram a BU, considerando apenas a medida de efeito entre a avaliação inicial e final, dois identificaram redução significativa da área da UV no final do seguimento^(45,48)^ e outro identificou taxa de cicatrização mais significativa^(49)^.

Alguns estudos incluídos^(26,31,34,37,39,42,43,50)^ fizeram a análise de fatores independentes associados à cicatrização e identificaram algumas evidências. A primeira foi o tempo de existência da UV com tempo de cicatrização^(26,50)^, ou seja, UV mais recentes cicatrizaram mais rápido. O tempo de existência da UV teve também relação direta com a taxa de cicatrização^(42,50)^. Outros encontraram que o tamanho da UV inicial influenciou no tempo de cicatrização, UV menores cicatrizaram mais rápido^(31,34,37,39,43)^. Um encontrou relação entre o tamanho inicial da UV e a taxa de cicatrização, UV menores cicatrizaram em maior proporção^(42)^.

### Eventos Adversos

Dos estudos incluídos, 17 relataram ocorrências de EA, sendo dez relacionados à bandagem de 4 camadas; seis com bandagem de curto estiramento; três com BU e bandagem de 2 camadas; dois com bandagem de 2 camadas coesivas e 3 camadas. A bandagem de quatro camadas foi a mais utilizada entre estudos que relataram os eventos e teve o maior número de eventos adversos relatados. Quanto ao total, o evento adverso mais citado foi o surgimento de nova úlcera, com 73 relatos.

### Risco de Viés

Dos 39 estudos, 28 (71,8%) eram ECR. Os ECR foram avaliados com a ROB-2 *tool* ^(20)^, quanto ao objetivo de análise estatística, de avaliação do efeito da atribuição à intervenção, efeito de intenção de tratar (ITT) e efeito por protocolo (PP). A ferramenta separa a avaliação de acordo com o tipo de efeito em estudo. A Figura 2 mostra os 14 (50%) ECR incluídos no estudo que foram realizados com ITT. Segundo a análise, seis (42,9%) estudos foram considerados de baixo risco de viés, três (21,4%) com algumas preocupações e cinco (35,7%) com alto risco de viés. Dos 14 (50%) ECR que realizaram análise PP, um (7,1%) foi avaliado com baixo risco de viés e 13 (92,9%) com alto risco de viés. Os estudos de coorte e caso-controle foram avaliados com a ROBINS-I *tool* ^(21)^. Nesta avaliação, os dez (25,6%) estudos de coorte e o único (2,5%) caso-controle apresentaram sério risco de viés.

**Figura 2:** Avaliação do risco de viés de ECR com análise por intenção de tratar.

**Fonte:** Elaborado pelos autores na ferramenta ROB-2, 2022.

### Síntese dos Resultados

Dos 39 estudos incluídos na RS, foi possível realizar a metanálise somente com 25 (64,1%). Conforme mencionado nos métodos, a metanálise foi organizada em subconjuntos, conforme os resultados apresentados. No subconjunto da taxa de cicatrização de UV, o gráfico *forest plot* (Figura 3) apresenta o número de úlceras cicatrizadas nos 25 estudos incluídos, organizados entre grupos de TC, comparados um a um. Destes, quatro (10,3%) utilizaram a BU e essa mostrou-se superior para o tratamento em apenas um estudo^(51)^. Este estudo comparou^(51)^ BU (n = 30) com cuidado usual (n = 30), demonstrando uma probabilidade de 6,5 mais chances de cicatrização com uso da BU (RR 6.50 – IC 95% 1.6 – 26.36)^(51)^. Em comparação com as demais TC, quanto ao número de úlceras cicatrizadas ao longo do seguimento, a BU mostrou-se equivalente no estudo que a comparou com bandagem de 2 camadas^(40)^ (RR 1.02 – IC 95% 0.92 – 1.14), naquele que a comparou com bandagem de 4 camadas^(31)^ (RR 0.88 IC 95% 0.64 – 1.22) e no que a comparou com bandagem de curto estiramento de 2 camadas^(17)^ (RR 1.20 IC 95% 0.41 – 3.51). Já no estudo que comparou a BU com outras TC^(17)^, essa foi inferior no número de UV cicatrizadas na comparação com compressão pneumática (RR 0.35 IC 95% 0.16 – 0.77), com Ulcer X (RR 0.35 IC 95% 0.16 – 0.77) e com bandagem de curto estiramento multicamadas (RR 0.34 IC 95% 0.16 – 0.74).

**Figura 3:** Metanálise do número de úlceras cicatrizadas comparado entre grupos de terapias compressivas.

**Fonte:** Elaborado pelos autores, 2024.

No subconjunto tempo para cicatrização da UV, 16 estudos foram incluídos. Entre os três que utilizaram a BU, não foi identificada superioridade de efeito desta TC no tempo para cicatrização da UV. No subconjunto diferença de área da UV, cinco estudos foram incluídos. Destes, quatro utilizaram a BU e essa mostrou-se com superioridade para o tratamento em apenas um estudo. Este estudo comparou BU (n = 9) com bandagem elástica de alta compressão (n = 9), tendo como resultado uma média de redução significativa da área da UV (–23.62, IC 95% –41.07 – –6.17)^(10)^. Entre as demais TC e cuidado usual, a BU mostrou-se equivalente para redução da área da UV ao longo do seguimento.

Nesta RS, a avaliação da heterogeneidade ficou limitada. Apenas cinco estudos compararam as mesmas terapias compressivas, bandagem de 2 camadas versus bandagem de 4 camadas (Figura 3). Quanto à avaliação de viés de publicação, mesmo que as buscas tenham atingido uma boa abrangência com inclusão de 25 estudos na metanálise, poucos compartilhavam das mesmas intervenções e da intervenção de interesse nesta RS, o que configurou uma amostra pequena, menor que dez. Portanto, não foi possível fazer avaliação do viés de publicação.

## DISCUSSÃO

Apesar da predominância de estudos no continente europeu, identificaram-se investigações em diversos países do mundo, reforçando que a UV é um problema de saúde pública^(2,3)^. Além disso, os resultados reforçam a maior incidência de IVC e UV em idosos e mulheres^(1,61,63)^. Dois estudos^(39,50)^ encontraram associação entre a idade e o tempo para cicatrização de UV, evidenciando que pessoas mais velhas tiveram suas úlceras cicatrizadas em um tempo maior que pessoas mais jovens.

Dentre as TC utilizadas, a bandagem 4 camadas foi a mais utilizada, seguida da BU, da bandagem 2 camadas e da bandagem de curto estiramento. O achado reforça as terapias de multicamadas como opção frequente, e em segundo lugar, as terapias inelásticas como a BU e a bandagem de curto estiramento^(5,6)^.

No que tange ao desfecho cicatrização, do total de estudos que utilizaram a BU em um de seus grupos, observou-se que a maioria foi realizada no Brasil, reforçando o argumento de ser uma TC comumente utilizada no país^(45,49)^ e, ao mesmo tempo, uma preocupação de pesquisadores brasileiros em buscar evidências para apoiar essa prática clínica. Por outro lado, apenas três eram ECR. Considerando a relevância deste tipo de delineamento para avaliação do efeito de uma intervenção^(64)^, tal achado reforça a importância da realização de ECR com avaliação do efeito da BU em pacientes com úlceras venosas, promovendo as melhores evidências.

Ainda referente à cicatrização, observou-se que não houve uniformidade na apresentação dos resultados e foram utilizadas diferentes mensurações do desfecho, impossibilitando a realização de metanálise abrangendo todos os estudos. Sugere-se que estudos que avaliam esse desfecho sejam realizados com base na área inicial e área final da UV, no tempo para cicatrização, e no percentual de UV cicatrizadas ao final do seguimento.

Quanto ao método de verificação das características da cicatrização, a planimetria digital e a fotografia foram as formas de medida mais frequentes. Embora o cálculo da área da UV na planimetria digital seja computadorizado, o traçado da UV na maioria dos estudos foi realizado manualmente, método tido como referência na literatura^(65)^.

Na metanálise, a BU foi superior para cicatrização comparada ao cuidado usual em um estudo^(51)^ e, em outro, com relação à atadura elástica de alta compressão^(10)^. Contrapondo os achados da presente investigação, uma RS^(12)^ identificou, com moderado grau de evidência, indiferença nas taxas de cicatrização das UV quando comparada a BU com outras TC.

Os resultados dos estudos encontrados que compararam TC com cuidado usual para o desfecho cicatrização reforçaram os achados descritos em duas metanálises^(5,6)^, as quais mencionam que o uso de algum tipo de compressão é superior a não utilização. Na comparação entre diferentes tipos de TC, foi observado que os achados se confirmam, ou seja, as bandagens de multicamadas são mais efetivas; as bandagens de 4 camadas mostraram-se superiores à bandagem de curto estiramento, mas equivalentes à bandagem de 2 camadas e as meias elásticas foram superiores à bandagem de curto estiramento^(17,28,30,32,34,37,43)^.

Embora os estudos incluídos identificassem superioridade em suas análises, provavelmente não há diferença significativa entre as TC; contudo, observa-se evidência do uso como superior ao cuidado usual. Possivelmente a justificativa se deve ao efeito terapêutico estar também relacionado às características da UV, ao autocuidado, à adesão e ao acesso ao tratamento e à tolerância à TC utilizada.

Esta RS não avaliou o efeito de todas as terapias. Apesar disso, salienta-se que embora outras TC com mais tecnologia sejam eficientemente superiores a BU, esta tem sido utilizada com a justificativa de baixo custo, embora não se tenha análise econômica para apoiar esta afirmação. Assim, configura-se como opção de tratamento para países com reduzidos recursos na área da saúde^(12)^.

Revisão integrativa da literatura que analisou estudos quanto aos tipos de TC em UV, enfatizando o uso da BU, identificou ampla defesa do uso da BU por sua ação curativa efetiva e pelos custos inferiores. Somado a isso, oito estudos incluídos na revisão indicaram o efeito positivo da BU no controle do edema, na redução da área e cicatrização das lesões, bem como na melhoria da qualidade de vida dos indivíduos^(66)^. Por outro lado, a BU pode exigir maior tempo para cicatrização comparada com a bandagem de multicamadas, visto que o seu mecanismo de ação depende da deambulação^(11,67)^.

Além das evidências quanto à efetividade da TC, ressalta-se que a escolha da terapia precisa considerar a gravidade da IVC^(3)^, o tamanho e o tempo de existência da UV, a circunferência da panturrilha e a mobilidade do tornozelo. Outro fato importante a ser considerado é o quanto o paciente se adapta à terapia utilizada e demonstra melhor adesão. Alguns estudos avaliaram conforto e/ou satisfação do paciente no uso da terapia^(31,32,35,37,42,55)^; a exemplo, cita-se estudo que identificou que conforto, dor na aplicação da terapia e facilidade de calçar sapatos melhoraram ao longo do tratamento; contudo, não foi encontrada diferença entre as TC utilizadas^(25)^.

Portanto, considerando a prevalência das UV, a diversidade e falta de resultados robustos nas evidências, sugere-se a realização de estudos de intervenção comparando diferentes tipos de TC para o tratamento da UV com análise de custo efetividade e com avaliações dos desfechos considerando o momento inicial e final do seguimento.

Observando os EA relatados nos estudos, chama a atenção que a dor aparece como evento bastante frequente, embora o desfecho esperado com o uso da TC seja a sua redução. Considerando o tipo de intervenção, observou-se que a BU esteve presente em 10% dos eventos relatados e a TC de 4 camadas, por ser proporcionalmente a mais utilizada, apresentou relatos em maior número. Considerando a gravidade e a relação dos EA com a intervenção, a maioria dos eventos relatados pode ser considerada leve e moderada, mas também associada à clínica da IVC e UV, tais como dor, maceração da pele e abertura de novas ulcerações.

Analisando-se a qualidade da evidência dos estudos incluídos, a maioria foi procedente de ECR, porém, com alto risco de viés. Tal resultado foi influenciado pela falta de informação sobre o cegamento na avaliação e análise dos resultados. A maioria dos estudos referiu que, devido à natureza do tratamento, não foi possível cegar os pacientes e profissionais que aplicaram a intervenção. Contudo, poucos mencionaram se houve cegamento do avaliador dos desfechos e/ou do profissional que realizou a análise dos resultados. Além disso, em algumas investigações, o avaliador dos desfechos foi o mesmo profissional que aplicou a intervenção, situação que compromete o grau de evidência do estudo, aumentando o risco de viés para avaliação do efeito da intervenção^(20)^. Considerando as avaliações realizadas nas pesquisas incluídas nessa RS, é importante destacar que o estudo que encontrou superioridade da BU sobre a atadura elástica de alta compressão para cicatrização da UV^(10)^ foi considerado com alto risco de viés devido à ausência de informação sobre o cegamento na avaliação do desfecho.

O alto grau de risco de viés nos estudos incluídos, o curto período de acompanhamento, o número limitado de pacientes, a diversidade de terapias e de avaliações de desfecho afetaram a análise dos resultados e a robustez das evidências. Achados esses são consistentes com RS de 2012, na qual a maioria dos ECR tinha amostras pequenas e risco incerto ou alto de viés^(5)^.

Diante disso, e considerando a homogeneidade na qualidade da evidência entre os estudos, avaliou-se a partir do sistema GRADE que os resultados encontrados por eles apresentaram moderado grau de evidência. Contudo, entende-se que as evidências de estimativa do efeito da TC na cicatrização podem ser modificadas por estudos futuros.

## LIMITAÇÕES

As limitações foram relacionadas à: diversidade na apresentação dos resultados pelos estudos incluídos, fato que não permitiu realizar metanálise de todos os estudos e avaliação de heterogeneidade; inclusão de estudos publicados somente em inglês, português e espanhol; limitação de acesso a alguns estudos na íntegra de forma gratuita.

## CONCLUSÃO

Embora a BU ainda seja utilizada de forma bastante frequente, não foram encontradas evidências de que a BU é mais efetiva para cicatrização de UV quando comparada com outras TC. Entretanto, ela mostra-se efetiva quando comparada ao cuidado usual. Assim, diante do cenário de oferta de bandagens sem classificação de compressão, como ocorre no Brasil, ainda a BU se mostra como a melhor opção terapêutica para tratamento de usuários com UV.

Considerando a variedade de TC no mercado, a escolha da TC conforme a gravidade da IVC e os poucos estudos com diferenças estatísticas entre as TC, destaca-se que a realização de ECR com análise de custo-efetividade pode contribuir na escolha terapêutica. Diante do contexto de ampla utilização de BU no Brasil, mais estudos ainda precisam ser realizados para avaliar a sua real efetividade.

Com relação à qualidade da evidência, a realização de algum método de cegamento nas avaliações do efeito das terapias, na análise dos resultados, faz-se fundamental, bem como a clara menção da forma de cegamento adotada. O não cumprimento deste quesito compromete a qualidade do estudo, impactando na redução de pontuação na análise de qualidade da evidência.

Os resultados dessa RS podem contribuir para a atuação dos profissionais de saúde ao apresentar evidências científicas que reforçam a superioridade do uso de qualquer TC em relação ao cuidado usual, contribuindo para a tomada de decisão e trazendo subsídios para discussão da oferta de insumos nos serviços de saúde.
